# Montelukast ameliorated pemetrexed-induced cytotoxicity in hepatocytes by mitigating endoplasmic reticulum (ER) stress and nucleotide oligomerization domain-like receptor protein 3 (NLRP3) activation

**DOI:** 10.1080/21655979.2022.2051689

**Published:** 2022-03-15

**Authors:** Zhengdong Fei, Lu Zhang, Lei Wang, Hui Jiang, Aiqin Peng

**Affiliations:** a Department of Ultrasound, Shuyang Hospital, the Affiliated Shuyang Hospital of Xuzhou Medical University, Suqian, Jiangsu, China; b Department of Radiology, Shuyang Hospital, the Affiliated Shuyang Hospital of Xuzhou Medical University, Suqian, Jiangsu, China

**Keywords:** Pemetrexed, Montelukast, hepatotoxicity, oxidative stress, endoplasmic reticulum stress

## Abstract

Pemetrexed (PEM) is an effective chemotherapeutic drug used for the treatment of clinical non-small-cell lung cancer (NSCLC) and is reported to induce severe hepatotoxicity. Exploring potential drugs which could counteract the side effects of PEM is of great clinical interest. Here, we aim to examine the beneficial effects of Montelukast, a novel anti-asthma drug, against PEM-induced cytotoxicity in hepatocytes, and to explore the underlying mechanism. We found that Montelukast reduces cytotoxicity of PEM in hepatocytes, confirmed by its increasing cell viability and reducing lactate dehydrogenase (LDH) release. In addition, Montelukast attenuated PEM-induced oxidative stress by reducing mitochondrial reactive oxygen species (ROS), increasing reduced glutathione (GSH), and downregulating NADPH oxidase 4 (NOX-4) expression. Importantly, Montelukast suppressed PEM-induced activation of the nucleotide oligomerization domain-like receptor protein 3 (NLRP3) inflammasome and mitigated endoplasmic reticulum (ER) stress by reducing NLRP3, growth arrest, and DNA damage-inducible protein 34 (GADD34), CEBP-homologous protein (CHOP), and also blocking the eukaryotic initiation factor 2 (eIF-2α)/activating transcription factor 4 (ATF4) signaling pathway. Lastly, we found that Montelukast inhibited the transcriptional activity of nuclear factor kappa-B (NF-κB). Montelukast exerted a protective action against PEM-induced cytotoxicity in hepatocytes by mitigating ER stress and NLRP3 activation.

## GRAPHICAL ABSTRACT

**Figure:**
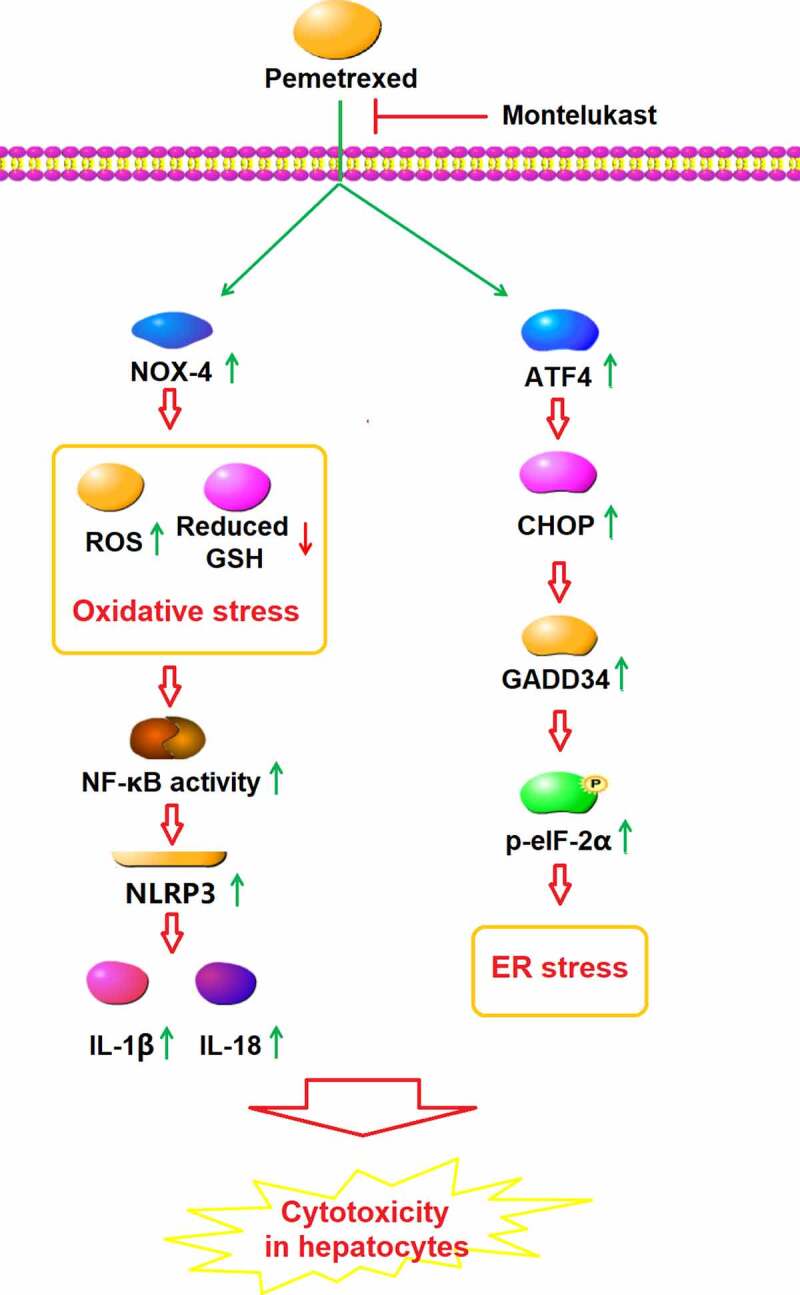

## Introduction

Amongst all types of malignant tumors in China, the incidence of lung cancer still ranks first and is increasing yearly [1]. The 5-year survival rate of lung cancer remains relatively low [2,3]. Currently, radiotherapy and chemotherapy are the main methods for the treatment of clinical non-small-cell lung cancer (NSCLC) patients, although issues including resistance and severe side effects keep rising during the process of treatment [4–7]. Pemetrexed (PEM) is an efficacious chemotherapeutic drug applied for the treatment of NSCLC, it is a novel inhibitor of folate metabolism based on methanol and fluorouracil [8]. PEM suppresses cellular replication and growth by interfering with the folate-dependent normal metabolic process. By targeting multiple enzymes, PEM stops cell division in the S phase by inhibiting the bioactivity of folate-dependent enzymes, leading to disturbances in nucleotide synthesis and folate metabolism [9,10]. Although PEM has shown promising anti-tumor efficacy in both clinical NSCLC patients and experimental animals [11,12], just like other chemotherapeutic drugs, it has been reported to have severe side effects, including hepatotoxicity, leukopenia, and neurotoxicity [13]. Recently, multiple mechanisms are reportedly involved in the toxicity of PEM against normal cells, including hepatocytes. Yan [14] reported that significant endoplasmic reticulum stress (ERS) in the hepatocytes could be induced by treatment with PEM. In addition, cytokine secretion is also reported to be an important side effect induced by PEM treatment [15]. Lastly, excessive production of ROS induced by PEM is another vital element that triggers cytotoxicity [16]. Therefore, there is an urgent need to explore a drug that can weaken the side effects of PEM.

Montelukast, a selective leukotriene receptor antagonist, has been developed by Merck and approved by the Food and Drug Administration (FDA) for the management of asthma [17,18]. In clinical practice, Montelukast exerts anti-asthma and anti-anaphylactic rhinitis effects by dilating bronchial smooth muscle and decreasing vascular permeability [19]. Montelukast has shown significant anti-inflammatory and anti-oxidative stress effects in both clinical and experimental treatments [20–22]. In the present study, we aim to investigate the beneficial effect of Montelukast against PEM- induced cytotoxicity in hepatocytes and the underlying mechanism to explore the potential therapeutic property of Montelukast against PEM-induced hepatotoxicity.

## Materials and methods

### Cell lines and treatments

The primary human LO-2 hepatocytes were purchased from ATCC (USA) and incubated with Dulbecco’s modified eagle medium (DMEM) containing 10% fetal bovine serum (FBS) at 37°C with 5% CO_2_. Cells were divided into four groups and incubated with pemetrexed (250 nM) and Montelukast (5, 10 μM) for 24 h.

### 3-(4, 5-dimethylthiazol-2-yl)-2, 5-diphenyl-tetrazolium bromide (MTT) assay

MTT was used to detect the cell viability of LO-2 hepatocytes. After treatment, the cells were exposed to 5 mg/mL MTT (10 μL) for 4 h. Subsequently, dimethylsulfoxide (DMSO, China) was used to dissolve formazan. Finally, the OD was read at 490 nm [23].

### LDH release measurement

The LDH assay kit (Roche, Switzerland) was used to detect the release of LDH from treated LO-2 hepatocytes. Briefly, the cells were centrifugated at 300 × g for 10 min and 60 µL supernatant was mixed with 30 µL LDH substrate solution. After incubation at 37°C for 30 min, the LDH levels were measured at 440 nm [24].

### Measurement of mitochondrial ROS

After treatment, cells were added with mixed 200 nM of MitoSOX Red (Invitrogen, California, USA) for 30 minutes. The confocal microscope (Olympus, Tokyo, Japan) was used to take images of the stained cells. Signals were analyzed using the software Image J (NIH, USA).

### The detection of reduced GSH

A commercial kit (Dojindo, USA) was applied to measure the level of reduced GSH in the treated human LO-2 hepatocytes. In brief, the cells were lysed and centrifugated, and further incubated with the coenzyme working solution for 2 hours. Finally, a microreader (Bio-Tek Instruments, Winooski, USA) was utilized to determine the absorbance at 405 nm.

### Real-time PCR analysis

The total RNAs were isolated from the treated human LO-2 hepatocytes using the TRIzol reagent (Invitrogen, California, USA) and were transformed into cDNA utilizing a commercial kit (Thermo Fisher Scientific, USA). Then, the transcribed cDNA was used for the quantitative real-time PCR with the Power SYBR Green Master Mix (Invitrogen, USA). The relative expression of genes was analyzed with the 2-^ΔΔct^ method with GAPDH utilized for normalization.

### Western blot assay

The treated human LO-2 hepatocytes were lysed to isolate proteins, which were subsequently loaded and separated with sodium dodecyl sulfate-polyacrylamide gel electrophoresis (SDS-PAGE). Then, the proteins were transferred onto the polyvinylidene difluoride (PVDF) membrane and incubated with 5% nonfat milk. The membrane was then incubated with primary antibody against NOX-4 (1:1000, ab133303, Abcam), NLRP3 (1:1000, ab263899, Abcam), p-eIF-2α (1:1000, ab169528, Abcam), ATF4 (1:1000, ab184909, Abcam), GADD34 (1:1000, ab236516, Abcam), CHOP (1:1000, ab11419, Abcam), NF-κB p65 (1:1000, ab288751, Abcam) or β-actin (1:1000, ab8226, Abcam), which was further incubated with an HRP-conjugated secondary antibody. Finally, the blots were developed with electrochemiluminescence (ECL) reagents and analyzed using Image J software [25].

### Enzyme-linked immunosorbent assay (ELISA)

The secretions of IL-1β and IL-18 were detected using ELISA kits (Abcam, Cambridge, USA). 50 μL of standard, control, and samples were added to each well and incubated overnight at 4°C. After that, a 50 μL antibody cocktail was added to each well and incubated for 30 min at room temperature. 100 μL of substrate solution was then added to each well and incubated for 20 min at room temperature, followed by a reaction with 50 μL of stop solution to each well. Absorbance was measured at 450 nm.

### Luciferase activity assay

The treated human LO-2 hepatocytes were planted in a 12-well plate and were transfected with 450 ng NF-κB promoter firefly luciferase reporter (Clontech, California, USA) for 24 h. After treatment, the Dual-Luciferase Reporter Assay System (Promega, Wisconsin, USA) was used to detect the activity of the reporter enzyme in the cellular extracts with a luminometer (Turner BioSystems, USA).

### Statistical analysis

Results were expressed as mean ± standard deviation and analyzed using the GraphPad Prism 7.0 software. Significant differences were analyzed by analyses of variance (ANOVA) method followed by Tukey’s post-hoc test. Significance was defined as P < 0.05.

## Results

To evaluate the benefits of Montelukast against PEM- induced lung injury, we established an *in vitro* model using PEM-stimulated human LO-2 hepatocytes. Our findings demonstrate that treatment with Montelukast alleviated PEM-induced oxidative stress, ER stress, activation of NLRP3 and NF-κB, indicating Montelukast as a promising therapeutic compound for the treatment of PEM-induced hepatotoxicity.

### Montelukast prevented PEM-induced cell death and release of LDH in human LO-2 hepatocytes

**Figure 1.**
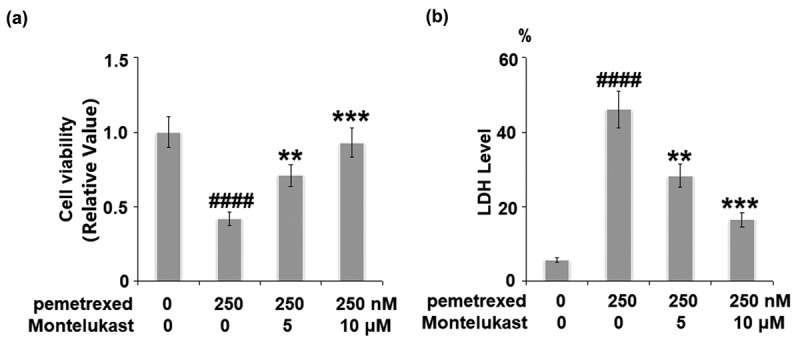
Montelukast prevented pemetrexed-induced cell viability and release of LDH in human LO-2 hepatocytes. Cells were incubated with pemetrexed (250 nM) and Montelukast (5, 10 μM) for 24 h. Cell viability and release of LDH was measured (####, P < 0.0001 vs. vehicle; **, ***, P < 0.01, 0.001 vs. pemetrexed treatment group).

The cell viability was remarkably suppressed by treatment with PEM, then dramatically elevated by the introduction of Montelukast (5, 10 μM) (Figure 1(a)). Compared to the control, the release of LDH in the PEM group was increased from 5.8% to 46.1%, which was decreased to 28.3% and 16.5% by 5 and 10 μM Montelukast, respectively (Figure 1(b)).

### Montelukast ameliorated PEM-induced oxidative stress

**Figure 2.**
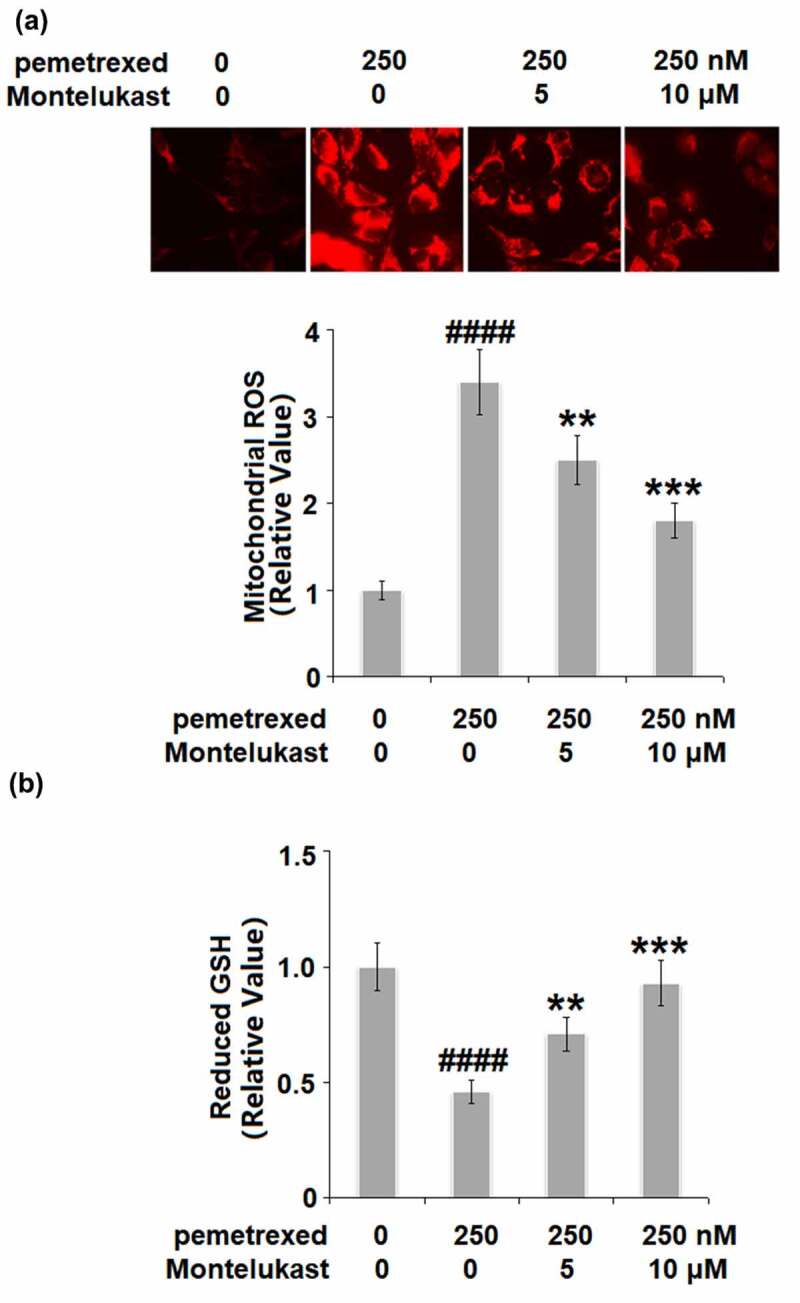
Montelukast ameliorated pemetrexed-induced oxidative stress. (a). Mitochondrial ROS; (b). The level of reduced GSH (####, P < 0.0001 vs. vehicle group; **, ***, P < 0.01, 0.001 vs. pemetrexed treatment group).

We further investigated the state of oxidative stress in the human LO-2 hepatocytes with different treatments. As shown in Figure 2(a), the mitochondrial ROS level in the human LO-2 hepatocytes was dramatically increased by PEM but greatly suppressed by Montelukast dose-dependently. In addition, PEM treatment inhibited the expression of reduced GSH (Figure 2(b)). However, the two doses of Montelukast pronouncedly elevated the expression of reduced GSH. These data indicate that PEM-induced oxidative stress in human LO-2 hepatocytes was obviously ameliorated by Montelukast.

### Montelukast reduced PEM-induced expression of NOX-4

**Figure 3.**
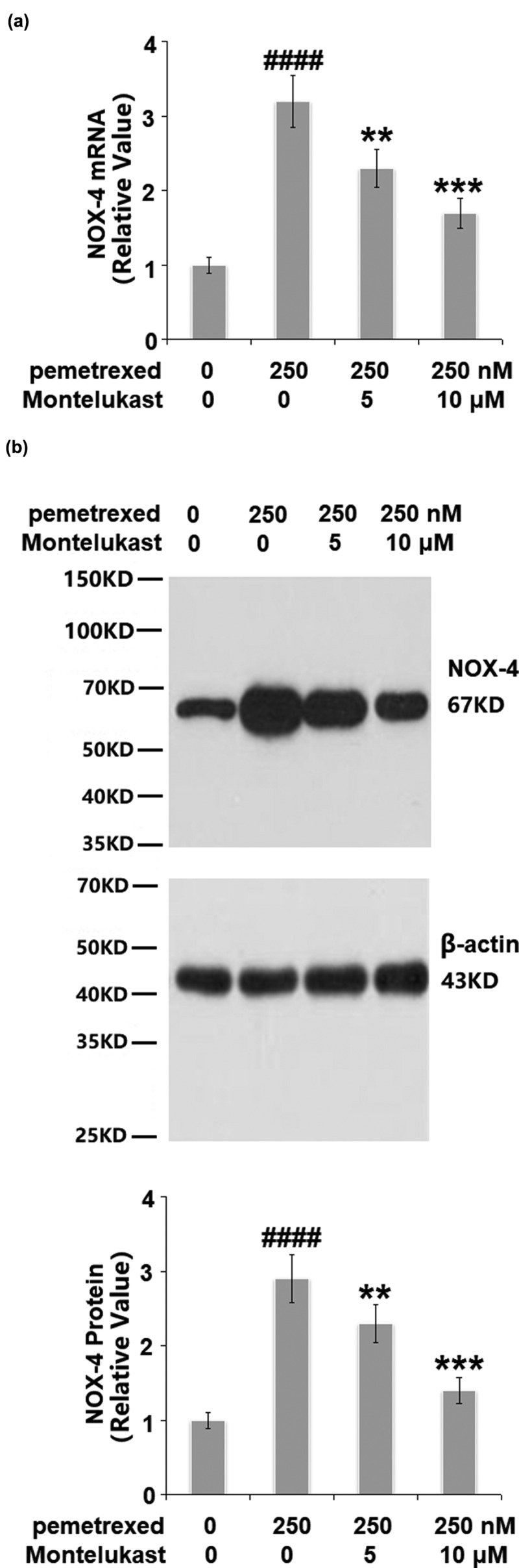
Montelukast reduced pemetrexed-induced expression of NOX-4. (a). mRNA of NOX-4; (b). Protein of NOX-4 (####, P < 0.0001 vs. vehicle; **, ***, P < 0.01, 0.001 vs. pemetrexed treatment group).

The expression of NOX-4 was detected to evaluate the oxidative state of the treated LO-2 hepatocytes. PEM induced a 3.6- fold increase of NOX-4 at the mRNA level, which was suppressed to approximately 2.5- and 1.6- fold (Figure 3). Similarly, the two doses of Montelukast reduced the protein level of NOX-4 to 2.3- and 1.5- fold, compared with a 3.1-fold increase induced by treatment with PEM. These results indicate that the oxidative state in the human LO-2 hepatocytes induced by PEM was alleviated by Montelukast.

### Montelukast alleviated PEM-induced activation of the NLRP3 inflammasome

**Figure 4.**
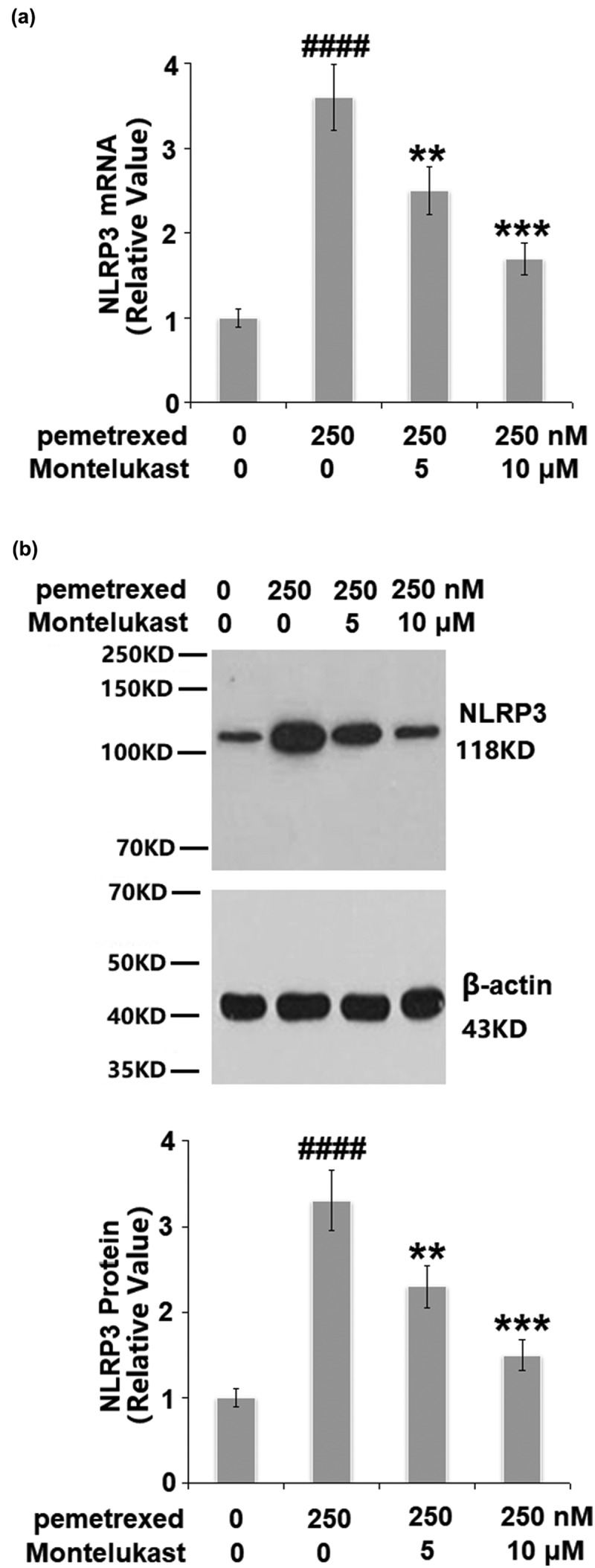
Montelukast suppressed pemetrexed-induced activation of NLRP3. (a). mRNA level of NLRP3; (b). Protein level of NLRP3 (####, P < 0.0001 vs. vehicle; **, ***, P < 0.01, 0.001 vs. pemetrexed treatment group).

**Figure 5.**
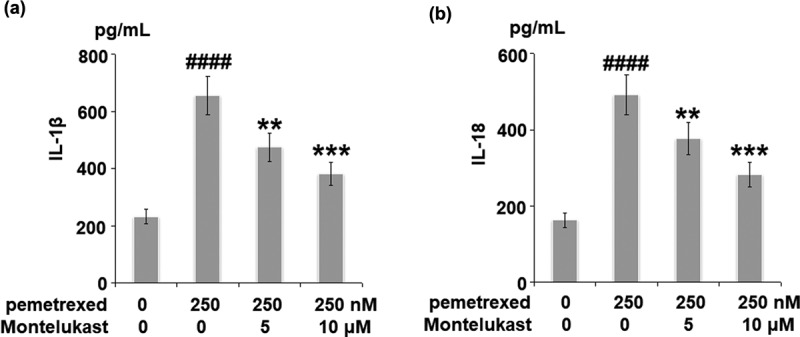
Montelukast suppressed pemetrexed-induced secretion of IL-1β and IL-18. (a). Secretions of IL-1β; (b). Secretions of IL-18 (####, P < 0.0001 vs. vehicle group; **, ***, P < 0.01, 0.001 vs. pemetrexed treatment group).

To assess the effects of Montelukast on the inflammation induced by PEM, the expression of NLRP3, and the secretion of IL-1β and IL-18 were investigated. As shown in Figure 4, the expression of NLRP3 was obviously upregulated by the treatment with PEM but significantly suppressed by Montelukast. Compared to the control, the concentration of IL-1β was enhanced from 232.5 to 655.8 pg/mL by PEM then reduced to 476.9 and 381.2 pg/mL by treatment with 5 and 10 μM Montelukast, respectively (Figure 5(a)). In addition, the concentration of IL-18 was elevated from 163.4 to 492.7 pg/mL, then inhibited to 377.1 and 282.5 pg/mL by treatment with 5 and 10 μM Montelukast, respectively (Figure 5(b)). These data show that the severe inflammation in human LO-2 hepatocytes induced by PEM was mitigated by Montelukast.

### Montelukast inhibited PEM-induced ER stress in human LO-2 hepatocytes

**Figure 6.**
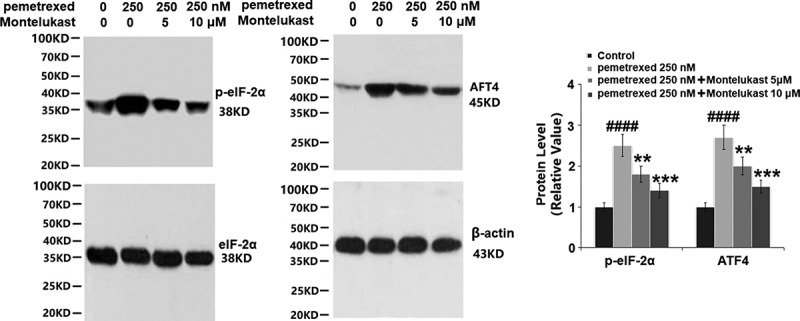
Montelukast inhibited pemetrexed-induced eukaryotic initiation factor-2α (eIF-2α)/ATF4. The expressions of p-eIF-2α and ATF4 (####, P < 0.0001 vs. vehicle group; **, ***, P < 0.01, 0.001 vs. pemetrexed treatment group).

**Figure 7.**
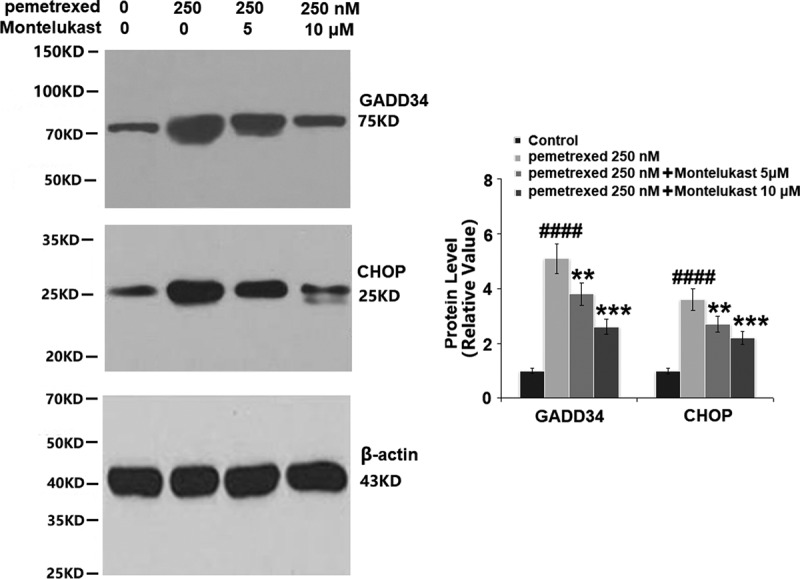
Montelukast prevented pemetrexed–induced expression of GADD34 and CHOP.(a). mRNA of GADD34 and CHOP; (b). Protein of GADD34 and CHOP (####, P < 0.0001 vs. vehicle group; **, ***, P < 0.01, 0.001 vs. pemetrexed treatment group).

To examine the roles of Montelukast on PEM-induced ER in human LO-2 hepatocytes, the expressions of ER-related proteins were detected following different treatments. As shown in Figure 6, p-eIF-2α and ATF4 were significantly up-regulated by treatment with PEM, then apparently down-regulated by the introduction of Montelukast. Additionally, the expressions of GADD34 and CHOP were dramatically increased by PEM but pronouncedly suppressed by the introduction of Montelukast (Figure 7). These findings indicate that PEM-induced ER stress in human LO-2 hepatocytes was obviously alleviated by Montelukast.

### Montelukast suppressed the activation of NF-κB

**Figure 8.**
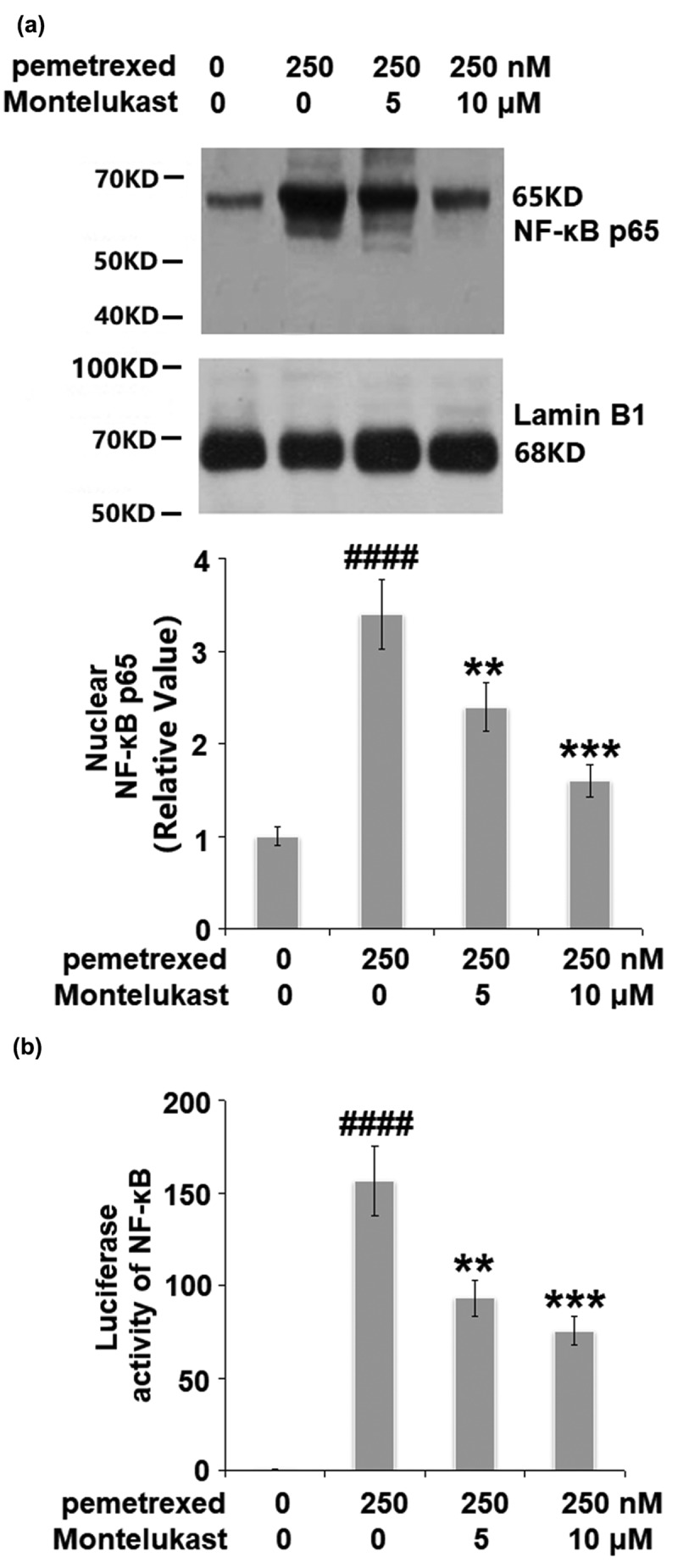
Montelukast suppressed the activation of NF-κB.(a). Nuclear levels of NF-κB p65; (b). Luciferase activity of NF-κB (####, P < 0.0001 vs. vehicle group; **, ***, P < 0.01, 0.001 vs. pemetrexed treatment group).

We further investigated the effect of Montelukast on the activated NF-κB signaling pathway induced by PEM. The nuclear level of NF-κB p65 was remarkably increased by PEM but significantly suppressed by the treatment with Montelukast. In addition, the increased luciferase activity of NF-κB (Figure 8(b)) induced by PEM was dramatically inhibited by Montelukast incubation. These data indicate that the activated NF-κB in human LO-2 hepatocytes induced by PEM was obviously inhibited by Montelukast.

## Discussion

Under a normal physiological state, the metabolism of ROS is maintained by the balance between the oxidative and anti-oxidative systems to keep the cellular functions. However, when severe injury is induced on hepatocytes by multiple external elements, such as lipopolysaccharides and chemotherapeutics, excessive ROS are released and accumulate to induce the state of oxidative stress, which contributes to further injury on hepatocytes [26,27]. The main components, including proteins, lipids, and DNA, can be damaged by oxidative stress by the release of excessive peroxides and free radicals [28,29]. Here, we report that severe cytotoxicity was induced by the treatment with PEM. By the introduction of Montelukast, the hepatocyte cytotoxicity was greatly mitigated, indicating a beneficial effect of Montelukast against PEM-treated hepatocytes. In addition, oxidative stress in the hepatocytes was dramatically activated by PEM, which was indicated by the elevation of ROS levels and decrease of the GSH level, as well as the up-regulation of NOX-4. We found that the state of oxidative stress was obviously ameliorated by the treatment with Montelukast, indicating an inhibitory effect of Montelukast against PEM-induced oxidative stress in the hepatocytes. The endoplasmic reticulum in the hepatocytes is the main site for drug metabolism, which is mainly mediated by the CYP450 enzyme system located on the membrane. However, when the metabolic balance in the endoplasmic reticulum is damaged, endoplasmic reticulum stress (ERS) is induced, ultimately contributing to the apoptosis of hepatocytes and hepatotoxicity [30]. ERS is a series of stress reactions induced by the activated unfolded protein response (UPR) and caspase 12-mediated apoptotic signaling pathway, which are triggered by the accumulation of misfolded and unfolded proteins in the lumen of the endoplasmic reticulum and calcium imbalance when the cells are impacted by stressors [31]. Multiple signaling pathways participate in the regulation of ERS, such as the inositol-requiring enzyme (IRE1) [32], pancreatic ER kinase (PERK) [33], and eIF-2α/ATF4 signaling pathways [34]. ERS is an important inducer of severe inflammation in the hepatocytes. It is reported that CHOP-dependent NLRP3 [35,36] and the NF-κB signaling pathway [37] can be activated by ERS, which further contributes to the excessive generation of inflammatory factors and secondary injury to hepatocytes. In the present study, we found that the eIF-2α/ATF4 signal pathway was significantly activated by PEM, which was reversed by the treatment with Montelukast, indicating that the ERS in the hepatocytes induced by PEM was greatly ameliorated by Montelukast. In addition, the elevated production of inflammatory factors, activated NLRP3 pathway, and activation of NF-κB in human LO-2 hepatocytes induced by PEM were dramatically reversed by Montelukast, indicating an inhibitory effect of Montelukast against severe inflammation induced by PEM. In our future work, an inhibitor of the eIF-2α/ATF4 signal pathway will be introduced to verify the impact of Montelukast on ERS. In addition, the specific target of Montelukast on the eIF-2α/ATF4 signal pathway will also be investigated to better understand the effects of Montelukast on ERS and the cytotoxicity in hepatocytes induced by PEM.

## Conclusion

Our data indicate that Montelukast exerted a protective effect against the PEM-induced cytotoxicity in hepatocytes by ameliorating ER stress and NLRP3 activation, shedding a light on the potential use of Montelukast to counter the side effects of PEM in clinics.

## Data Availability

Requests for data and materials should be addressed to the corresponding author
